# Wealth-related inequalities in the coverage of reproductive, maternal, newborn and child health interventions in 36 countries in the African Region

**DOI:** 10.2471/BLT.19.249078

**Published:** 2020-04-08

**Authors:** Fernando C Wehrmeister, Cheikh Mbacké Fayé, Inácio Crochemore M da Silva, Agbessi Amouzou, Leonardo Z Ferreira, Safia S Jiwani, Dessalegn Y Melesse, Martin Mutua, Abdoulaye Maïga, Tome Ca, Estelle Sidze, Chelsea Taylor, Kathleen Strong, Liliana Carvajal-Aguirre, Tyler Porth, Ahmad Reza Hosseinpoor, Aluisio J D Barros, Ties Boerma, on the behalf of the Countdown to 2030 for Women’s, Children’s and Adolescents’ Health regional collaboration in sub-Saharan Africa

**Affiliations:** a International Center for Equity in Health, Federal University of Pelotas, Rua Marechal Deodoro, 1160, 3 piso, Pelotas, Brazil.; b African Population and Health Research Centre, West Africa Regional Office, Dakar, Senegal.; c Department of International Health, Johns Hopkins Bloomberg School of Public Health, Baltimore, United States of America (USA).; d Centre for Global Public Health, University of Manitoba, Canada.; e African Population and Health Research Center, Central Office, Nairobi, Kenya.; f Health Information System, West African Health Organization, Bobo-Dioulasso, Burkina Faso.; g Department of Data and Analytics, World Health Organization, Geneva, Switzerland.; h Department of Maternal, Newborn, Child, and Adolescent Health and Aging, World Health Organization, Geneva, Switzerland.; i Division of Data, Analytics, Planning and Monitoring, United Nations Children’s Fund, New York, USA.

## Abstract

**Objective:**

To investigate whether sub-Saharan African countries have succeeded in reducing wealth-related inequalities in the coverage of reproductive, maternal, newborn and child health interventions.

**Methods:**

We analysed survey data from 36 countries, grouped into Central, East, Southern and West Africa subregions, in which at least two surveys had been conducted since 1995. We calculated the composite coverage index, a function of essential maternal and child health intervention parameters. We adopted the wealth index, divided into quintiles from poorest to wealthiest, to investigate wealth-related inequalities in coverage. We quantified trends with time by calculating average annual change in index using a least-squares weighted regression. We calculated population attributable risk to measure the contribution of wealth to the coverage index.

**Findings:**

We noted large differences between the four regions, with a median composite coverage index ranging from 50.8% for West Africa to 75.3% for Southern Africa. Wealth-related inequalities were prevalent in all subregions, and were highest for West Africa and lowest for Southern Africa. Absolute income was not a predictor of coverage, as we observed a higher coverage in Southern (around 70%) compared with Central and West (around 40%) subregions for the same income. Wealth-related inequalities in coverage were reduced by the greatest amount in Southern Africa, and we found no evidence of inequality reduction in Central Africa.

**Conclusion:**

Our data show that most countries in sub-Saharan Africa have succeeded in reducing wealth-related inequalities in the coverage of essential health services, even in the presence of conflict, economic hardship or political instability.

## Résumé

**Objectif:**

Déterminer si les pays d'Afrique subsaharienne sont parvenus à réduire l'impact des inégalités de richesse sur la prise en charge des interventions de santé reproductives, maternelles et infantiles.

**Méthodes:**

Nous avons analysé des données d'enquêtes menées dans 36 pays regroupé en sous-régions (Afrique centrale, Afrique de l'Est, Afrique australe et Afrique de l'Ouest). Au moins deux enquêtes devaient dater d'après 1995. Nous avons calculé l'indice de prise en charge composite en fonction de plusieurs paramètres essentiels d'interventions de santé maternelle et infantile. Nous avons également adopté l'indice de richesse divisé en quintiles, du plus pauvre au plus nanti, afin d'étudier l'impact des inégalités de richesse dans cette prise en charge. Nous avons quantifié les tendances par rapport au temps, en calculant l'évolution annuelle moyenne de l'indice à l'aide de la régression des moindres carrés pondérés. Enfin, nous avons évalué le risque attribuable à la population pour mesurer la contribution de la richesse à l'indice de prise en charge.

**Résultats:**

Nous avons remarqué des différences considérables entre les quatre régions, avec un indice médian de prise en charge composite allant de 50,8% en Afrique de l'Ouest à 75,3% en Afrique australe. Les inégalités liées à la richesse étaient très répandues dans toutes les sous-régions; les plus fortes ont été observées en Afrique de l'Ouest, les plus faibles en Afrique australe. Le revenu absolu n'est pas un indicateur valable car nous avons constaté une meilleure prise en charge dans la sous-région d'Afrique australe (environ 70%) que dans celles d'Afrique centrale et d'Afrique de l'Ouest (environ 40%) pour des revenus équivalents. La majorité des inégalités de prise en charge liées à la richesse ont diminué en Afrique australe, et nous n'avons trouvé aucune preuve de réduction des inégalités en Afrique centrale.

**Conclusion:**

Nos données montrent que la plupart des pays d'Afrique subsaharienne ont réussi à atténuer les inégalités de prise en charge liées à la richesse pour les services de santé essentiels, même en cas de conflit, de difficultés économiques ou d'instabilité politique.

## Resumen

**Objetivo:**

Investigar si los países del África subsahariana han logrado reducir las desigualdades relacionadas con la riqueza en la cobertura de las intervenciones sanitarias relativas a la salud reproductiva, materna, neonatal e infantil.

**Métodos:**

Se analizaron los datos de las encuestas de 36 países, agrupados en las subregiones de África central, oriental, meridional y occidental, en los que se habían realizado por lo menos dos encuestas desde 1995. Se calculó el índice compuesto de cobertura, en función de los parámetros esenciales de las intervenciones sanitarias relativas a la salud maternoinfantil. Se adoptó el índice de riqueza, dividido en quintiles de más pobres a más ricos, para investigar las desigualdades de cobertura relacionadas con la riqueza. Se cuantificaron las tendencias con el tiempo al calcular el cambio anual medio del índice por medio de una regresión por mínimos cuadrados ponderados. También se calculó el riesgo atribuible a la población para medir la contribución de la riqueza al índice de cobertura.

**Resultados:**

Se observaron grandes diferencias entre las cuatro regiones, con un índice compuesto medio de cobertura que oscilaba entre el 50,8 % para el África occidental y el 75,3 % para el África meridional. Las desigualdades relacionadas con la riqueza prevalecían en todas las subregiones y eran mayores en el África occidental y menores en el África meridional. Los ingresos absolutos no eran un factor de predicción de la cobertura, ya que se observó una mayor cobertura en las subregiones del sur (alrededor del 70 %) en comparación con las subregiones central y occidental (alrededor del 40 %) para los mismos ingresos. Las desigualdades de cobertura relacionadas con la riqueza se redujeron en mayor medida en el África meridional, y no encontramos pruebas de reducción de la desigualdad en el África central.

**Conclusión:**

Estos datos muestran que la mayoría de los países del África subsahariana han logrado reducir las desigualdades relacionadas con la riqueza en la cobertura de los servicios sanitarios esenciales, incluso en presencia de conflictos, dificultades económicas o inestabilidad política.

## ملخص

الغرض التحقق مما إذا كانت الدول الأفريقية في جنوب الصحراء الكبرى قد نجحت في الحد من حالات عدم المساواة المتعلقة بالثروة في تغطية التدخلات الصحية المتعلقة بالصحة الإنجابية، وصحة الأم، وصحة المواليد، وصحة الأطفال.

الطريقة قمنا بتحليل بيانات المسح من 36 دولة، ثم تجميعها في مناطق وسط وشرق وجنوب وغرب إفريقيا الفرعية، والتي تم فيها إجراء مسحين على الأقل منذ عام 1995. وقمنا بحساب مؤشر التغطية المركبة، وهي وظيفة لمعاملات التدخل الصحي الأساسية للأم والطفل. قمنا بانتهاج مؤشر الثروة، وقسمناه إلى الفئات الخمسية من الأشد فقراً إلى الأكثر غنى، للتحقيق في حالات عدم المساواة المتعلقة بالثروة في التغطية. وقمنا بقياس الاتجاهات مع مرور الوقت عن طريق حساب متوسط ​​التغيير السنوي في المؤشر باستخدام انحدار المربعات الصغرى المرجح. قمنا بحساب المخاطر التي تعزى إلى السكان لقياس مساهمة الثروة في مؤشر التغطية.

النتائج لاحظنا وجود اختلافات كبيرة بين المناطق الأربع، مع متوسط ​​للمؤشر المركب للتغطية من 50.8% في غرب إفريقيا إلى 75.3% في جنوب أفريقيا. كانت حالات عدم المساواة المتعلقة بالثروة سائدة في كل المناطق الفرعية، وكانت في أعلى درجاتها في غرب أفريقيا، وأدنى درجاتها في جنوب أفريقيا. لم يكن الدخل المطلق عاملاً للتنبؤ بالتغطية، حيث لاحظنا تغطية أعلى في المناطق الجنوبية (حوالي 70%) مقارنة بالمناطق الفرعية الوسطى والغربية (حوالي 40%) لنفس الدخل. تقلصت حالات عدم المساواة في التغطية المتعلقة بالثروة، بأكبر قدر ممكن في جنوب أفريقيا، بينما لم نجد دليلاً على الحد من عدم المساواة في وسط أفريقيا.

**الاستنتاج:**

تُظهر بياناتنا أن معظم الدول الأفريقية في جنوب الصحراء الكبرى قد نجحت في الحد من حالات عدم المساواة المتعلقة بالثروة في تغطية الخدمات الصحية الأساسية، حتى في وجود الصراع، أو الصعوبات الاقتصادية، أو عدم الاستقرار السياسي **. **

## 摘要

**目的:**

旨在调查撒哈拉以南的非洲国家是否成功减少了在生育、孕产妇、新生儿和儿童健康干预措施覆盖方面呈现出的与财富有关的不平等现象。

**方法:**

我们分析了来自中非、东非、南非和西非地区的 36 个国家的调查数据，这些国家自 1995 年以来已至少进行了两次调查。我们计算了综合覆盖指数，这是一种妇幼健康干预基本参数函数。我们采用财富指数，从最贫困到最富有共划分了五个等级，以调查在覆盖范围方面与财富相关的不平等现象。我们使用最小二乘加权回归法计算指数的年平均变化，从而量化其随时间的变化趋势。我们计算了人群归因风险度来衡量财富对覆盖指数的贡献。

**结果:**

我们发现这四个地区之间存在巨大差异，综合覆盖指数中位数从西非的 50.8%，到南非的 75.3%。与财富有关的不平等现象普遍存在于非洲各个次区域，其中西非的不平等程度最高，南非的不平等程度最低。我们观察到，在相同的收入水平下，南部地区（约 70%）的覆盖率高于中部和西部地区（约 40%），因此绝对收入并非覆盖率的预测指标。与财富相关的不平等现象在非洲南部减少最多，而在中非，我们没有发现不平等现象减少的迹象。

**结论:**

我们的数据显示，即使存在冲突、经济困难或政治动荡问题，大多数撒哈拉以南的非洲国家在基本卫生服务覆盖方面成功地减少了与财富相关的不平等现象。

## Резюме

**Цель:**

Изучить, удалось ли странам Африки, расположенным южнее Сахары, успешно сократить неравенство в охвате мероприятиями по охране репродуктивного здоровья, материнства, здоровья новорожденных и детского здравоохранения, связанное с уровнем доходов.

**Методы:**

Авторы проанализировали данные опросов в 36 странах, сгруппированных в субрегионы Восточной, Западной, Центральной и Южной Африки, в которых в период с 1995 года было проведено по меньшей мере два опроса. Авторы рассчитали составной индекс охвата как функцию существенных параметров мероприятий по охране здоровья матери и ребенка. За основу был взят показатель благосостояния, разбитый на квинтили от самых бедных до самых богатых, позволяющий изучить неравенство в охвате мероприятиями, связанное с различиями в уровне доходов. Авторы выполнили количественную оценку тенденций по времени посредством расчета среднегодового изменения показателя с использованием средневзвешенной регрессии по методу наименьших квадратов. Авторы рассчитали популяционный риск, чтобы измерить зависимость показателя охвата мероприятиями от уровня дохода.

**Результаты:**

Было отмечено значительное различие между четырьмя регионами: средний составной индекс охвата составил от 50,8% в Западной Африке до 75,3% в Южной Африке. Неравенство, связанное с уровнем доходов, присутствовало во всех субрегионах и было сильнее всего выражено в странах Западной Африки и слабее всего выражено в странах Южной Африки. Абсолютный доход не позволял прогнозировать уровень охвата мероприятиями по охране здоровья, поскольку авторы наблюдали более высокий уровень охвата (около 70%) в Южной Африке по сравнению с уровнем охвата в Центральном и Западном субрегионах (около 40%) при том же уровне дохода. Неравенство, связанное с различиями в уровне дохода, значительно уменьшилось в Южной Африке, тогда как в Центральной Африке снижения неравенства не отмечается.

**Вывод:**

Данные показывают, что большинству стран Африки, расположенных южнее Сахары, удалось сократить неравенство в охвате основными видами медицинских услуг, связанное с уровнем доходов, несмотря на наличие конфликтов, тяжелой экономической ситуации или политической нестабильности.

## Introduction

Reaching all women and children with essential reproductive, maternal, newborn and child health interventions is a critical part of universal health coverage, and is represented by the Global Strategy for Women’s, Children’s and Adolescents’ Health 2016–2030.^1^ The millennium development goals focused on national improvements and not within-country inequalities, even though initiatives such as the Countdown to 2015 Collaboration began tracking inequalities in coverage.^2^ However, the sustainable development goals and the associated global and country-specific strategies continue to emphasize the importance of women’s, children’s and adolescents’ health with a focus on reducing all dimensions of inequality.^3^

Globally, sub-Saharan Africa has the highest mortality rates for women during pregnancy and childbirth, for children and for adolescents, as well as the lowest coverage for many maternal, newborn and child health interventions.^4^ Economic status, measured by wealth of the household, is often one of the most critical factors affecting coverage of such interventions, with large gaps between the poorest and wealthiest households. A study published in 2011 based on 28 sub-Saharan African countries quantified the impact of wealth on inequalities in intervention coverage: in 26 of the 28 countries, within-country wealth-related inequality accounted for more than one quarter of the intervention coverage gap (the difference between full coverage, i.e. 100%, and the actual coverage).^5^ These findings illustrate the importance of the analysis of coverage trends by wealth to improve the targeting of health programmes.

We investigated the extent to which sub-Saharan African countries have succeeded in reducing wealth-related inequalities in maternal, newborn and child health intervention coverage. We calculated a composite coverage index, measured its changes over time and performed between-country comparisons to highlight subregional patterns and identify countries that have shown remarkable progress in reducing wealth-related inequalities. These analyses were conducted as part of four workshops organized and funded by the Countdown to 2030 for Women’s, Children’s and Adolescents’ Health programme, and attended by teams of analysts from public health institutions and ministries of health from 38 African countries in 2018.

## Methods

### Survey data

We performed our analysis on secondary data acquired from 127 national surveys conducted in 36 countries in which at least 2 surveys had been conducted since 1995. Surveys were either Demographic and Health Surveys or Multiple Indicator Cluster Surveys, which allowed us to compare standard indicators with time. We grouped the countries into four subregions according to the United Nations Population Division classification^6^: Central Africa (6 countries, 18 surveys), East Africa (11 countries, 41 surveys), Southern Africa (5 countries, 15 surveys) and West Africa (14 countries, 54 surveys; Table 1).

**Table 1:** Survey data used to calculate composite coverage index of reproductive, maternal, newborn and child health interventions in sub-Saharan African countries, 1996–2016

Country	Source	Year	Composite coverage index, % (SE)
**Central Africa**			
Cameroon	DHS	1998	39.7 (1.7)
Cameroon	DHS	2004	47.1 (1.1)
Cameroon	DHS	2011	47.6 (1.1)
Cameroon	MICS	2014	51.5 (1.2)
Chad	DHS	1996	18.6 (1.1)
Chad	DHS	2004	15.9 (1.2)
Chad	MICS	2010	19.7 (0.9)
Chad	DHS	2014	28.0 (0.9)
Congo	DHS	2005	51.6 (1.2)
Congo	DHS	2011	57.9 (1.0)
Congo	MICS	2014	56.1 (0.9)
Democratic Republic of the Congo	DHS	2007	41.7 (1.2)
Democratic Republic of the Congo	MICS	2010	43.9 (0.9)
Democratic Republic of the Congo	DHS	2013	47.1 (0.8)
Gabon	DHS	2000	46.3 (0.9)
Gabon	DHS	2012	58.1 (0.8)
Sao Tome and Principe	DHS	2008	68.1 (1.2)
Sao Tome and Principe	MICS	2014	73.6 (0.9)
**East Africa**			
Burundi	DHS	2010	56.1 (0.6)
Burundi	DHS	2016	62.6 (0.5)
Comoros	DHS	1996	46.0 (1.5)
Comoros	DHS	2012	51.7 (1.3)
Ethiopia	DHS	2000	16.3 (0.9)
Ethiopia	DHS	2005	24.7 (0.9)
Ethiopia	DHS	2011	35.1 (1.2)
Ethiopia	DHS	2016	45.1 (1.2)
Kenya	DHS	1998	57.9 (0.9)
Kenya	DHS	2003	52.4 (1.0)
Kenya	DHS	2008	59.1 (0.9)
Kenya	DHS	2014	70.4 (0.5)
Madagascar	DHS	1997	36.8 (1.3)
Madagascar	DHS	2003	43.5 (1.7)
Madagascar	DHS	2008	49.8 (1.0)
Malawi	DHS	2000	55.4 (0.7)
Malawi	DHS	2004	58.5 (0.6)
Malawi	DHS	2010	70.2 (0.4)
Malawi	MICS	2013	75.2 (0.4)
Malawi	DHS	2015	77.0 (0.4)
Mozambique	DHS	1997	40.8 (2.5)
Mozambique	DHS	2003	56.8 (1.0)
Mozambique	DHS	2011	54.6 (1.1)
Mozambique	DHS	2015	61.2 (1.4)
Rwanda	DHS	2000	33.2 (0.5)
Rwanda	DHS	2005	38.6 (0.5)
Rwanda	DHS	2010	63.5 (0.7)
Rwanda	DHS	2014	67.7 (0.5)
United Republic of Tanzania	DHS	1996	58.6 (1.1)
United Republic of Tanzania	DHS	1999	60.7 (2.2)
United Republic of Tanzania	DHS	2004	58.8 (1.0)
United Republic of Tanzania	DHS	2010	59.6 (0.9)
United Republic of Tanzania	DHS	2015	62.3 (0.9)
Uganda	DHS	2000	44.5 (0.9)
Uganda	DHS	2006	50.5 (0.8)
Uganda	DHS	2011	58.3 (0.7)
Uganda	DHS	2016	65.1 (0.5)
Zambia	DHS	1996	59.0 (0.9)
Zambia	DHS	2001	59.8 (0.9)
Zambia	DHS	2007	62.4 (1.0)
Zambia	DHS	2013	69.5 (0.7)
**Southern Africa**			
Eswatini	DHS	2006	78.1 (0.7)
Eswatini	MICS	2010	78.2 (0.7)
Eswatini	MICS	2014	83.3 (1.0)
Lesotho	DHS	2004	62.8 (0.9)
Lesotho	DHS	2009	68.6 (1.0)
Lesotho	DHS	2014	75.3 (0.9)
Namibia	DHS	2000	69.0 (1.1)
Namibia	DHS	2006	75.1 (1.0)
Namibia	DHS	2013	77.0 (0.7)
South Africa	DHS	1998	75.7 (0.6)
South Africa	DHS	2016	75.2 (1.1)
Zimbabwe	DHS	2005	57.5 (0.8)
Zimbabwe	DHS	2010	63.6 (0.9)
Zimbabwe	MICS	2014	75.9 (0.6)
Zimbabwe	DHS	2015	73.1 (1.1)
**West Africa**			
Benin	DHS	1996	41.7 (1.3)
Benin	DHS	2001	47.1 (1.0)
Benin	DHS	2006	45.8 (0.7)
Benin	DHS	2011	51.3 (0.7)
Benin	MICS	2014	48.1 (0.7)
Burkina Faso	DHS	1998	27.2 (1.2)
Burkina Faso	DHS	2003	36.8 (1.1)
Burkina Faso	DHS	2010	54.6 (0.8)
Côte d’Ivoire	DHS	1998	39.0 (2.0)
Côte d’Ivoire	DHS	2011	43.6 (1.2)
Côte d’Ivoire	MICS	2016	47.9 (1.0)
Gambia	MICS	2010	59.8 (0.7)
Gambia	DHS	2013	61.5 (0.7)
Ghana	DHS	1998	45.3 (1.0)
Ghana	DHS	2003	53.5 (1.0)
Ghana	DHS	2008	58.8 (1.0)
Ghana	MICS	2011	62.5 (0.8)
Ghana	DHS	2014	65.3 (0.9)
Guinea	DHS	1999	37.2 (1.2)
Guinea	DHS	2005	39.4 (1.4)
Guinea	DHS	2012	39.9 (1.2)
Liberia	DHS	2007	49.4 (1.5)
Liberia	DHS	2013	60.3 (1.0)
Mali	DHS	2001	29.3 (1.1)
Mali	DHS	2006	39.3 (0.9)
Mali	MICS	2009	39.9 (0.6)
Mali	DHS	2012	45.2 (1.1)
Mali	MICS	2015	39.6 (0.9)
Mauritania	MICS	2011	48.7 (0.8)
Mauritania	MICS	2015	49.4 (1.1)
Niger	DHS	1998	25.5 (1.3)
Niger	DHS	2006	28.9 (1.2)
Niger	DHS	2012	45.4 (1.0)
Nigeria	DHS	1999	37.4 (1.5)
Nigeria	DHS	2003	32.0 (1.5)
Nigeria	MICS	2007	35.1 (1.1)
Nigeria	DHS	2008	36.5 (0.9)
Nigeria	MICS	2011	41.9 (1.0)
Nigeria	DHS	2013	37.7 (1.0)
Nigeria	MICS	2016	35.9 (0.7)
Senegal	DHS	2005	45.5 (0.8)
Senegal	DHS	2010	51.5 (0.9)
Senegal	DHS	2012	51.3 (1.4)
Senegal	DHS	2014	54.1 (1.2)
Senegal	DHS	2015	55.2 (1.2)
Senegal	DHS	2016	56.5 (1.3)
Senegal	DHS	2017	61.9 (0.9)
Sierra Leone	DHS	2008	47.7 (1.1)
Sierra Leone	MICS	2010	62.1 (0.7)
Sierra Leone	DHS	2013	66.4 (0.9)
Sierra Leone	DHS	2017	71.0 (0.7)
Togo	DHS	1998	33.4 (1.1)
Togo	MICS	2010	45.1 (1.0)
Togo	DHS	2013	52.1 (1.1)

DHS: Demographic and Health Survey; MICS: Multiple Indicator Cluster Survey; SE: standard error.

### Composite coverage index

We calculated the composite coverage index (percentage) of maternal, newborn and child health interventions, a weighted function of essential maternal and child health intervention indicators representing the four-stage continuum of care (family planning, antenatal care and delivery, child immunization and disease management), defined as:^7^^–^^9^

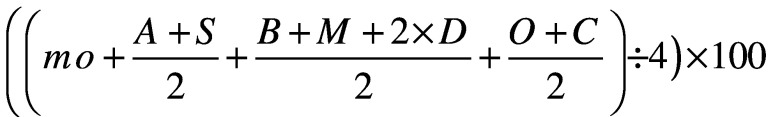
(1)where the variables represent the proportion of: women aged 15–49 years of age in need of contraception and had access to modern methods to modern contraceptive methods (*FPmo*), at least four antenatal care visits (*A*) and a skilled birth attendant (*S*); children aged 12–23 months who received tuberculosis immunization by Bacillus Calmette–Guérin (*B*), measles immunization (*M*) and three doses of diphtheria–tetanus–pertussis immunization (or pentavalent vaccine) (*D*); and children younger than 5 years of age who received oral rehydration salts for diarrhoea treatment (*O*) and care for suspected acute respiratory infection (*C*). The index is a robust single measure of the coverage of such interventions and is particularly suitable for examining broad patterns of inequality; it has also been reported to correlate well with health-related indicators such as the mortality of children younger than 5 years of age and stunting prevalence.^9^

### Wealth-related inequalities

We adopted the wealth index to examine inequalities, which is based on a principal component analysis of dwelling and household assets. The wealth index is weighted according to the assets in urban and rural places of residence, and then divided into quintiles; the first quintile represents the poorest 20% in the population and the fifth quintile represents the wealthiest 20%.^10^^,^^11^ We then calculated the predicted absolute income attributed to each within-country wealth distribution quintile^12^ using: data from the International Center for Equity in Health database,^13^ acquired from surveys conducted in low- and middle-income countries; gross domestic product data adjusted for purchasing parity (extracted from the World Bank);^14^ and income inequality data from the World Income Inequality Database.^15^ By using absolute income data, we expand the capability of the wealth index to explore inequalities within countries and over time.^12^

We used the software Stata, version 15 (StatCorp, College Station, Texas), to describe wealth-related inequalities according to the most recent survey for each country. We provide the calculated composite coverage index and its standard error, based on a binomial distribution, for the entire population and for the poorest and wealthiest quintiles within each country. For the relationship between absolute income and composite coverage index, we considered each quintile as independent and performed a locally weighted scatterplot smoothing regression.

### Time trends

We analysed time trends in the composite coverage index for the entire population, and for the poorest and wealthiest groups within each country and subregion by calculating the average annual absolute change (percentage points) in the composite coverage index. We used a least-squares regression weighted by the standard error of the composite coverage index estimate for each year in country-specific analyses. We used a multilevel approach for subregional analysis and considered the country as the level-two regression variable.

### Contribution of wealth

To investigate the contribution of wealth to composite coverage index, or to quantify the level of health intervention coverage that would be achieved if wealth-related inequalities were eliminated, we calculated the population attributable risk. The World Health Organization (WHO) Handbook on Health Inequality Monitoring defines population attributable risk (in percentage points), or absolute inequality, as the coverage gap in the wealthiest quintile subtracted from the coverage gap in the entire population.^16^ Alternatively, we define population attributable risk in terms of coverage, that is, the coverage in the entire population subtracted from the coverage in the wealthiest quintile. Mathematically equivalent to the WHO definition, we believe our definition in terms of coverage (instead of coverage gap) is simpler. 

Relative inequality, or population attributable risk percentage, can be calculated as population attributable risk expressed as a percentage of the composite coverage index within the entire population. We calculated the population attributable risk and its percentage for each country for two different time periods, using the oldest survey up until 2010 and the most recent survey from 2011 onwards.

### Ethics

All survey data are publicly available, and all ethical aspects were the responsibility of the relevant agencies and countries.

## Results

### Coverage and inequalities

We observed a median composite coverage index (median year: 2014) of 60.8% from the most recent surveys of sub-Saharan African countries until 2016. We noted large differences between the four regions, with a composite coverage index ranging from 50.8% for West Africa to 75.3% for Southern Africa. Between countries, we calculated the lowest value of the index of 28.0% for Chad and the highest of 83.3% for Eswatini (Table 1). 

**Fig. 1:**
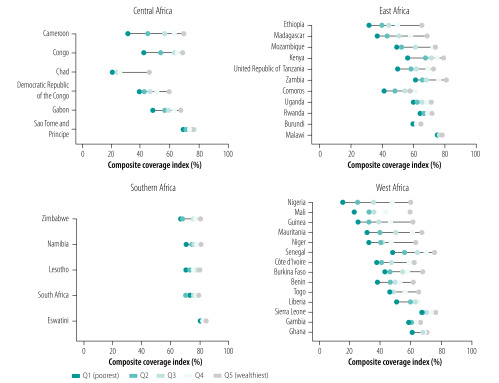
Current levels of wealth-related inequalities in health intervention coverage, sub-Saharan Africa, 1996–2016

In terms of within-country inequalities, Fig. 1 highlights the higher levels of coverage among the wealthier groups in all countries. We note that the highest levels of coverage, as well as the lowest within-country inequalities, are observed for Southern African countries. We observed much higher inequalities between rich and poor within the other three subregions, especially in West Africa; however, such subregional inequalities vary greatly within each subregion, ranging from, for instance, very high inequality in Nigeria (West) and Ethiopia (East) to virtually no difference in intervention coverage between rich and poor in Ghana (West) and Malawi (East).

**Fig. 2:**
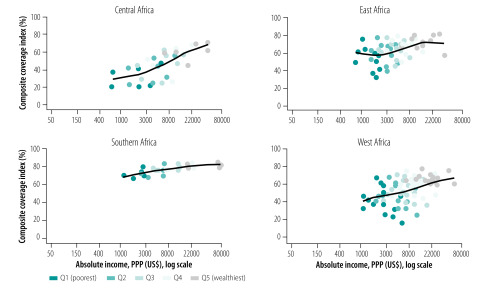
Composite coverage index as a function of absolute income, sub-Saharan Africa, 1996–2016

PPP: purchasing power parity; US$: United States dollars.

We also note how the association between predicted absolute income and composite coverage index differs by subregion (Fig. 2); at comparable income levels, coverage is considerably higher in East and Southern Africa than in Central and West Africa. For instance, at an income level of 1000 United States dollars, coverage is about 40% in Central and West Africa, around 60% in East Africa and over 70% in Southern Africa. We also observed marked differences within the subregions; the coverage index in Southern Africa is relatively constant with absolute income, while health intervention coverage increases almost linearly with predicted absolute income in both West and Central Africa subregions (Fig. 2).

### Time trends

**Fig. 3:**
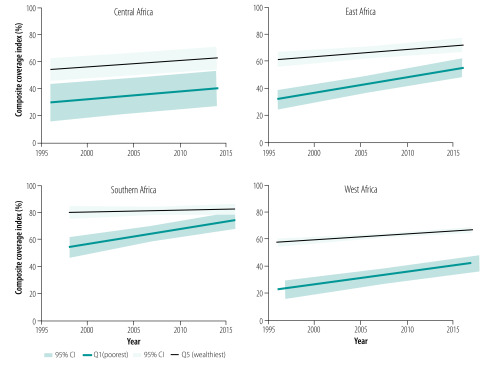
Changes in composite coverage index with time for the poorest (Q1) and wealthiest (Q5) groups, sub-Saharan Africa, 1996–2016

We depict the evolution of the composite coverage index in the poorest and wealthiest groups, by subregion, in Fig. 3 (see also Table 2). In Southern Africa, the average annual increase in coverage among the poorest (1.86; 95% confidence interval (CI): 1.11 to 2.60) was higher than among the wealthiest (0.02; 95% CI: −0.86 to 0.89), reducing the inequality between rich and poor. Inequalities by income have been statistically not significant since 2013 in Southern Africa. Inequalities have also been reducing in the last two decades in East Africa, as the average annual increase in coverage among the poorest (1.21; 95% CI: 0.77 to 1.64) was higher than among the wealthiest (0.56; 95% CI: 0.14 to 0.97); however, a substantive gap between the two groups remains. If the average annual increase over the last two decades continues, we predict that the discrepancy in coverage between rich and poor will be eliminated before 2030. Inequalities in West Africa has been substantially reduced, but by 2015 the estimated gap was still around 25 percentage points. Finally, we observed no evidence of a reduction in wealth-related inequalities in Central Africa.

CI: confidence interval.

**Table 2:** Annual average change in composite coverage index of reproductive, maternal, newborn and child health interventions in sub-Saharan African countries, 1996–2016,

Country	Average annual change, percentage points (95% CI)		
	Entire population	Q1	Q5
**Central Africa**	0.6 (0.4 to 0.9)	0.8 (−0.2 to 1.7)	0.6 (0.2 to 1.0)
Cameroon	0.6 (0.6 to 0.8)	0.0 (−0.3 to 0.3)	0.5 (0.3 to 0.8)
Chad	0.5 (0.4 to 0.7)	0.6 (0.4 to 0.8)	0.4 (0.2 to 0.6)
Congo	0.5 (0.2 to 0.8)	−0.02 (−0.6 to 0.5)	0.8 (0.1 to 1.4)
Democratic Republic of Congo	0.9 (0.5 to 1.4)	0.9 (0.2 to 1.6)	0.3 (−0.4 to 1.1)
Gabon	1.0 (0.8 to 1.2)	1.2 (0.9 to 1.6)	0.5 (0.0 to 1.0)
Sao Tome and Principe	0.9 (0.4 to 1.4)	0.3 (−0.7 to 1.2)	0.1 (−1.5 to 1.7)
**East Africa**	1.0 (0.7 to 1.3)	1.2 (0.8 to 1.6)	0.6 (0.1 to 1.0)
Burundi	1.1 (0.8 to 1.3)	1.3 (0.8 to 1.8)	0.7 (0.2 to 1.2)
Comoros	0.4 (0.1 to 0.6)	0.6 (0.2 to 0.9)	−0.4 (−0.8 to 0.0)
Ethiopia	1.8 (1.6 to 2.0)	1.3 (1.1 to 1.5)	1.8 (1.5 to 2.2)
Kenya	1.0 (0.9 to 1.1)	0.9 (0.7 to 1.1)	0.7 (0.5 to 0.9)
Madagascar	1.2 (0.9 to 1.5)	1.1 (0.7 to 1.5)	0.5 (0.2 to 0.9)
Malawi	1.6 (1.5 to 1.7)	1.9 (1.7 to 2.0)	0.8 (0.6 to 0.9)
Mozambique	0.5 (0.3 to 0.7)	1.1 (0.8 to 1. 5)	0.1 (−0.1 to 0.3)
Rwanda	2.7 (2.6 to 2.8)	2.6 (2.4 to 2.7)	2.2 (2.0 to 2.4)
United Republic of Tanzania	0.2 (0.1 to 0.3)	0.3 (0.0 to 0.5)	−0.1 (−0.3 to 0.1)
Uganda	1.3 (1.2 to 1.5)	1.4 (1.2 to 1.6)	0.6 (0.4 to 0.8)
Zambia	0.7 (0.5 to 0.8)	0.9 (0.7 to 1.1)	0. 3 (0.1 to 0.5)
**Southern Africa**	0.6 (0.1 to 1.0)	1.9 (1.1 to 2.6)	0.0 (−0.9 to 0.9)
Eswatini	0.6 (0.3 to 0.9)	1.3 (0.9 to 1.8)	0.2 (−0.6 to 1.0)
Lesotho	1.3 (1.0 to 1.5)	2.1 (1.6 to 2.5)	0.5 (−0.1 to 1.1)
Namibia	0.6 (0.4 to 0.8)	1.0 (0.6 to 1.5)	0.0 (−0.4 to 0.4)
South Africa	0.0 (−0.2 to 0.1)	0.5 (0.2 to 0.8)	−0.2 (−0.5 to 0.2)
Zimbabwe	1.9 (1.7 to 2.1)	2.5 (2.1 to 3.0)	1.6 (1.1 to 2.2)
**West Africa**	0.8 (0.6 to 1.1)	1.0 (0.9 to 1.2)	0.5 (0.3 to 0.6)
Benin	0.3 (0.2 to 0.5)	0.3 (0.1 to 0.6)	−0.1 (−0.3 to 0.0)
Burkina Faso	2.3 (2.1 to 2.6)	2.0 (1.7 to 2.3)	1.4 (1.1 to 1.8)
Côte d’Ivoire	0.5 (0.3 to 0.8)	0.8 (0.3 to 1.3)	−0.1 (−0.5 to 0.2)
Gambia	0.6 (−0.1 to 1.2)	1.4 (0.4 to 2.4)	−0.4 (−1.8 to 1.0)
Ghana	1.2 (1.1 to 1.4)	1.7 (1.5 to 2.0)	0.4 (0.1 to 0.8)
Guinea	0.2 (−0.1 to 0.5)	0.1 (−0.2 to 0.5)	0.2 (−0.1 to 0.6)
Liberia	1.8 (1.2 to 2.4)	2.4 (1.5 to 3.3)	−0.5 (−1.5 to 0.5)
Mali	0.7 (0.5 to 0.9)	0.4 (0.2 to 0.7)	0.2 (−0.0 to 0.4)
Mauritania	0.2 (−0.5 to 0.9)	−0.3 (−1.2 to 0.7)	1.3 (0.2 to 2.4)
Niger	1.5 (1.3 to 1.7)	1.2 (0.9 to 1.5)	0.7 (0.4 to 1.0)
Nigeria	0.1 (−0.1 to 0.2)	0.0 (−0.1 to 0.2)	−0.5 (−0.7 to −0.3)
Senegal	1.2 (1.0 to 1.4)	1.1 (0.9 to 1.4)	1.0 (0.8 to 1.3)
Sierra Leone	1.9 (1.7 to 2.2)	2.2 (1.8 to 2.6)	1.5 (1.0 to 2.0)
Togo	1.2 (1.0 to 1.4)	1.4 (1.1 to 1.6)	0.8 (0.8 to 1.1)

CI: confidence interval; Q1: poorest quintile; Q5, wealthiest quintile.

We provide the annual average change in composite coverage index during 1995–2016 for each country in Table 2. Calculated for the entire population of each country, we observed statistically significant (*P* < 0.05) increases in health intervention coverage in all 36 countries except Gambia, Guinea, Mauritania, Nigeria and South Africa. We observed the largest average annual increases in coverage index in Burkina Faso (2.3 percentage points), Rwanda (2.7 percentage points) and Sierra Leone (1.9 percentage points).

In examining data for the poorest and wealthiest quintiles, we note that the coverage index statistically significantly increased among the poorest in 30 countries; among the wealthiest, coverage index increased in only 20 countries. The annual average annual change was greater in the poorest quintile than in the wealthiest quintile in 31 countries, although the 95% CIs overlapped for all countries. We note there were five countries in which wealth-related inequalities increased (Cameroon, Congo, Ethiopia, Guinea and Mauritania). We observed either very small increases or else decreases in the coverage index among the poorest in four of these countries. In Ethiopia, the average annual increase in coverage index among the poorest (1.3 percentage points) was outpaced by that among the wealthiest (1.8 percentage points).

### Impact of wealth 

We calculated the population attributable risk, that is, the contribution of wealth to the composite coverage index, in Table 3 for two separate periods. The population attributable risk declined considerably from the first to the second period: we note that the median population attributable risk and population attributable risk percentage declined from 16.5 to 10.9 percentage points and from 36.0% to 16.9%, respectively. 

**Table 3:** Changing contribution of wealth to composite coverage index of reproductive, maternal, newborn and child health interventions in sub-Saharan African countries, 1996–2016

Country	First period coverage (up until 2010)				Second period coverage (from 2011 onwards)			
	Entire population %	Q5 %	PAR^a^	PAR%^b^	Entire population %	Q5 %	PAR^a^	PAR%^b^
**Central Africa**								
Cameroon	39.7	58.0	18.3	46.0	51.5	69.0	17.5	34.0
Chad	18.6	38.5	19.9	106.9	28.0	45.3	17.3	62.0
Congo	51.6	62.9	11.4	22.0	56.1	68.6	12.5	22.3
Democratic Republic of Congo	41.7	57.1	15.5	37.1	47.1	59.1	12.1	25.6
Gabon	46.3	55.4	9.2	19.8	58.1	61.6	3.5	6.1
Sao Tome and Principe	68.1	74.4	6.3	9.2	73.6	75.0	1.4	1.9
**East Africa**								
Burundi	56.1	62.0	5.9	10.5	62.6	66.0	3.4	5.5
Comoros	46.0	63.1	17.1	37.2	51.7	56.9	5.1	9.9
Ethiopia	16.3	38.1	21.8	133.3	45.1	65.3	20.2	44.8
Kenya	57.9	72.9	15.0	25.9	70.4	80.0	9.6	13.7
Madagascar	36.8	62.4	25.6	69.5	49.8	68.3	18.6	37.3
Malawi	55.4	69.1	13.7	24.8	77.0	78.6	1.7	2.2
Mozambique	40.8	66.0	25.2	61.8	61.2	74.5	13.2	21.6
Rwanda	33.2	44.6	11.5	34.6	67.7	71.4	3.7	5.5
United Republic of Tanzania	58.6	74.2	15.6	26.6	62.3	72.9	10.6	17.0
Uganda	44.5	63.6	19.0	42.7	65.1	71.7	6.6	10.1
Zambia	59.0	75.8	16.8	28.5	69.5	81.2	11.7	16.8
**Southern Africa**								
Eswatini	78.1	82.9	4.8	6.1	83.3	85.2	1.8	2.2
Lesotho	62.8	75.0	12.2	19.5	75.3	79.8	4.4	5.9
Namibia	69.0	82.0	13.0	18.9	77.0	81.1	4.2	5.4
South Africa	75.7	82.9	7.2	9.5	75.2	79.9	4.7	6.3
Zimbabwe	57.5	70.8	13.3	23.1	73.1	81.7	8.6	11.7
**West Africa**								
Benin	41.7	62.4	20.7	49.6	48.1	59.2	11.1	23.1
Burkina Faso	27.2	50.0	22.8	83.7	54.6	68.3	13.7	25.1
Côte d’Ivoire	39.0	63.2	24.2	62.0	47.9	64.2	16.3	34.1
Gambia	59.8	68.4	8.6	14.3	61.5	67.1	5.6	9.1
Ghana	45.3	62.1	16.8	37.1	65.3	69.8	4.5	7.0
Guinea	37.2	58.6	21.4	57.5	39.9	61.6	21.8	54.7
Liberia	49.4	67.5	18.0	36.5	60.3	64.6	4.3	7.1
Mali	29.3	57.5	28.2	96.1	39.6	59.6	20.1	50.7
Mauritania	48.7	61.9	13.1	26.9	49.4	67.2	17.7	35.9
Niger	25.5	54.2	28.7	112.7	45.4	63.4	18.0	39.7
Nigeria	32.0	62.7	30.7	96.0	35.9	60.3	24.4	68.0
Senegal	45.5	61.6	16.1	35.4	61.9	75.7	13.8	22.3
Sierra Leone	47.7	61.7	14.0	29.3	71.0	77.4	6.4	9.0
Togo	33.4	53.9	20.5	61.5	52.1	65.7	13.6	20.6

PAR, population attributable risk; Q5, wealthiest quintile.

^a^ PAR = health intervention coverage in wealthiest quintile less that in total population.

^b^ PAR% = PAR as a percentage of composite coverage index for the entire population.

**Fig. 4:**
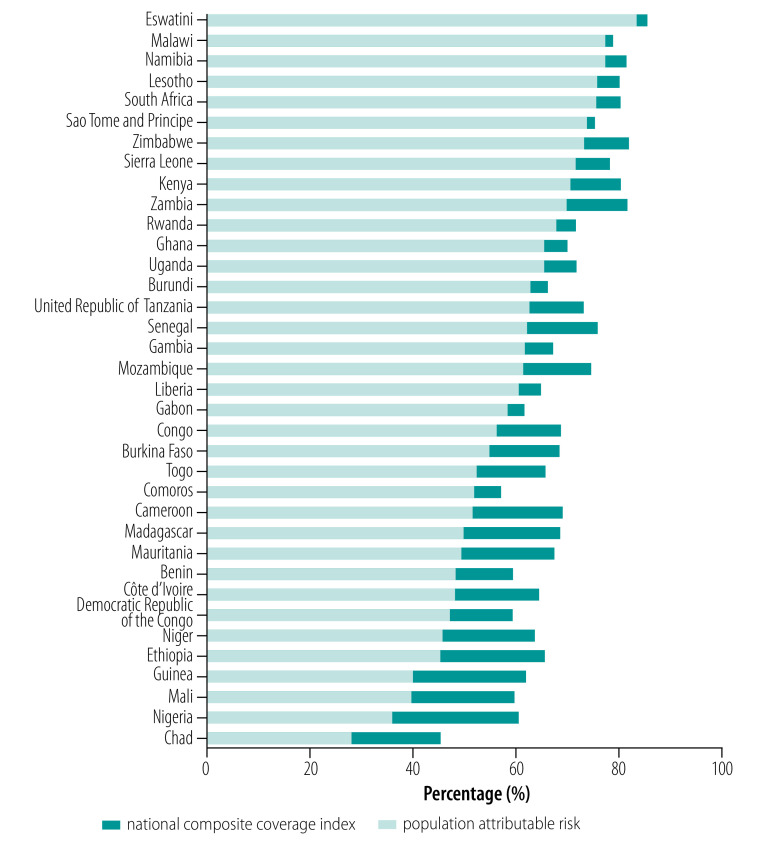
Composite coverage index achievable if wealth-related inequalities were eliminated, sub-Saharan Africa, 1996–2016

We show the potential for improvement in national coverage of reproductive, maternal, newborn and child health interventions in Fig. 4 by plotting the coverage that could be achieved if the entire population experienced the same level of coverage as the wealthiest subgroup. That is, we extended the composite coverage index as measured for the total population from the most recent survey by the population attributable risk calculated for the second period (Table 3). The four countries with the largest values of population attributable risk as calculated for the second period (Ethiopia, Guinea, Mali and Nigeria; Table 3) all had very low levels of national coverage (Table 1), but could achieve an improvement of 20 percentage points or more in national coverage if within-country wealth-related inequality was eliminated. In contrast, countries with a national coverage index of more than 70% typically have a small population attributable risk; however, some countries in this group (e.g. Kenya and Zimbabwe) would still see an improvement of approximately 10 percentage points.

## Discussion

We obtained encouraging results from our investigation; not only were many countries successful in improving coverage of essential reproductive, maternal, newborn and child health interventions, but they also succeeded in significantly reducing wealth-related inequalities in coverage during the last two decades. This progress was achieved without explicit focus on within-country inequalities. Even though our study was not designed to quantify the effects of context, policies or specific programmes on inequalities in coverage, we can provide several insights that are relevant to current efforts to achieve 100% coverage by 2030.

First, our data reveal very distinct subregional patterns in sub-Saharan Africa. Southern Africa has already eliminated much inequality and, if trends in East Africa continue, this region will also eliminate much of the remaining inequality in the coming decade. The Central and West African subregions were characterized by large wealth-related inequalities in coverage and slow progress to reduce these. Most countries in these subregions are currently not predicted to reduce wealth-related inequalities by 2030.^4^^,^^8^

Second, large differences in income or wealth do not necessarily translate into large differences in health intervention coverage. Income inequalities, measured by the Gini index, are considerably larger in Southern African countries than elsewhere in sub-Saharan Africa.^17^ The higher levels of health service utilization in Southern Africa could be a result of higher levels of education compared with countries in other sub-Saharan Africa regions.^18^

Third, our data show that coverage appears to stagnate at about 80%, irrespective of wealth; this stagnation may be partly the result of measurement challenges associated with some coverage indicators for which the exact denominator is difficult to measure (e.g. women in need of family planning and sick children in need of treatment). Greater efforts are also needed to identify and understand the populations that do not or cannot access these health interventions, for example, tracking the poorest 10% or ethnic minorities by targeted population surveys.^19^^,^^20^

Fourth, we observe considerable heterogeneity within the subregions, in terms of both national levels of coverage and wealth-related inequalities. In East Africa, Malawi and Rwanda have succeeded in reducing wealth-related inequalities; however, although Ethiopia has made progress in coverage, we observed no reduction in inequality. In West Africa, while Sierra Leone was one of the best performers in terms of both increased coverage and reduced inequality, we do not observe progress in either indicator in Guinea and Nigeria. Studies have identified several success factors: equitable policies for essential services, increased health expenditure per capita and strong implementation of national programmes in Malawi;^21^^,^^22^ and policies on human resources, health service delivery, health information systems and financing in Rwanda.^23^^–^^25^ A full analysis of factors contributing to reduced wealth-related inequalities requires an in-depth assessment of drivers of change and stagnation over a broader set of countries.

Fifth, our macro-level analyses provide insights into the effects of humanitarian emergencies on the coverage of essential health interventions. We observed rapid improvements in coverage in Liberia and Sierra Leone after prolonged periods of armed conflicts until the Ebola epidemic struck during 2013–2016. However, our analysis of the 2017 Demographic and Health Survey results in Sierra Leone showed that the composite coverage index continued to increase, and wealth-related inequalities continued to decrease. Our preliminary analysis of health intervention coverage using the 2016 Malaria Indicator Survey (MIS) in Liberia^26^ (which does not have data on family planning) and the 2018 Demographic and Health Survey in neighbouring Guinea also provide evidence of continued improvement (except in immunization). We therefore suggest that improvements in essential health intervention coverage are still possible during or shortly after epidemic situations. Other analysis has shown that strong and fast recovery in coverage is possible following conflicts,^27^ but large wealth-related inequalities persist in conflict-affected countries.^28^ Our analysis for Central Africa, where protracted political instability is greatest (Cameroon, Chad and Democratic Republic of Congo), shows that the wealthiest and poorest groups experienced increases in health intervention coverage at the same pace, that is, no reduction in wealth-related inequality. Poor access to primary health-care services for women and children and forced displacement are likely factors in Central Africa, and may also contribute to the large inequalities observed in other subregions. For example, the current conflict in northern Nigeria may be exacerbating existing wealth-related inequalities.^16^

Sixth, although the poorest quintile experienced lower health intervention coverage than the wealthiest quintile in every country, the patterns of inequality are different. In Chad, Ethiopia and Niger, the wealthiest quintile experiences much higher coverage than the other four quintiles, a pattern called top inequality.^29^ However, in countries such as Congo, Gabon and Kenya, the poorest quintile is far behind the other four quintiles, referred to as bottom inequality. These patterns should guide policy-makers in their prioritization of targeted or general health intervention approaches.

Our analysis should be interpreted in the light of several limitations. First, we used data from 36 countries in which at least two national surveys had been conducted since 1995, representing 75% (36/48) of the African countries and 89.4% (964 006 029/1 078 306 520) of the total population in sub-Saharan Africa. Several countries had not conducted a survey in the last 5 years, limiting our ability to assess subregional developments since 2015.^6^ Second, we used the composite coverage index as a measure of health intervention coverage, but that may have concealed differential inequalities in specific indicators such as family planning, immunization or delivery care; however, multiple standalone indicators would complicate the comparative assessment at country and regional levels. Third, a limitation is our use of a relative measure of socioeconomic position, that is, wealth index. This index is influenced by the choice of variables included; as most of the wealthiest individuals live in urban areas, the index could be reflecting urban–rural inequalities.^11^ Between-country comparisons using the wealth index are also misleading. Fourth, our results are not weighted by country population, even though we aimed to identify subregional patterns. However, we believe the unweighted results allow us to highlight the large between-country differences.

Addressing the wealth-related inequalities in coverage of essential health interventions remains a high-priority public health issue. A thorough analysis of factors contributing to wealth-related inequalities, as well as health intervention programmes targeting specific groups within a population, are required. However, our analyses demonstrate that countries can reduce wealth-related inequalities even in the presence of conflict, economic hardship or political instability.
